# Plasma and Nanomaterials: Fabrication and Biomedical Applications

**DOI:** 10.3390/nano9010098

**Published:** 2019-01-14

**Authors:** Nagendra Kumar Kaushik, Neha Kaushik, Nguyen Nhat Linh, Bhagirath Ghimire, Anchalee Pengkit, Jirapong Sornsakdanuphap, Su-Jae Lee, Eun Ha Choi

**Affiliations:** 1 Plasma Bioscience Research Center, Applied Plasma Medicine Center, Department of Electrical and Biological Physics, Kwangwoon University, Seoul 01897, Korea; nhatlinhusth@gmail.com (N.N.L.); ghimirebhagi@hotmail.com (B.G.); un_chaleep@hotmail.com (A.P.); jirakwangwoon@gmail.com (J.S.); 2 Department of Life Science, Hanyang University, Seoul 04763, Korea; neha.bioplasma@gmail.com

**Keywords:** plasma, nanomaterials, nanomaterial synthesis, plasma liquid Interactions, non-thermal plasma, biomedical applications

## Abstract

Application of plasma medicine has been actively explored during last several years. Treating every type of cancer remains a difficult task for medical personnel due to the wide variety of cancer cell selectivity. Research in advanced plasma physics has led to the development of different types of non-thermal plasma devices, such as plasma jets, and dielectric barrier discharges. Non-thermal plasma generates many charged particles and reactive species when brought into contact with biological samples. The main constituents include reactive nitrogen species, reactive oxygen species, and plasma ultra-violets. These species can be applied to synthesize biologically important nanomaterials or can be used with nanomaterials for various kinds of biomedical applications to improve human health. This review reports recent updates on plasma-based synthesis of biologically important nanomaterials and synergy of plasma with nanomaterials for various kind of biological applications.

## 1. Introduction

**Figure 1:**
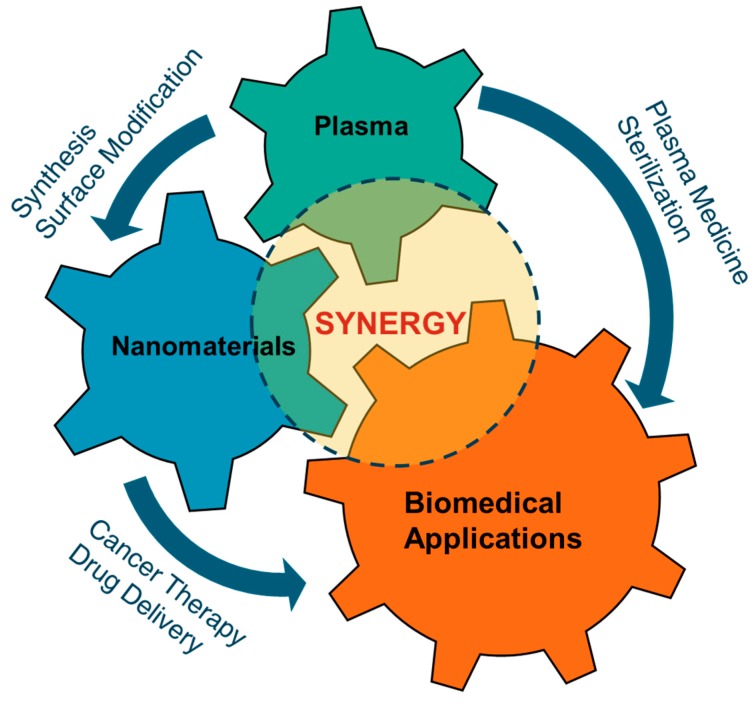
The synergistic relationship among plasmas, nanomaterials and their biomedical applications.

In recent years, nanomaterials have received great attention due to their exclusive characteristics compared to their bulk counterparts. With extremely small size and high surface area, nanomaterials demonstrate great biological activities in the human body. Nanomaterials play crucial roles in biomedicine, with a wide range of applications such as drug delivery, cancer therapy or bioimaging. Nevertheless, our current understanding of nanomaterials’ behaviors in human health is still inadequate. Previous reports have claimed that nanomaterials could induce dangerous effects in living organisms. A reasonable explanation for this concern is that conventional chemical approaches for nanomaterial synthesis require toxic oxidants or reductants, which are essential for nanoparticle formation and stabilization. Therefore, an alternative toxic-chemical-free synthesis is important for nanotechnology development for biomedical applications. Currently, plasma technology is gaining great attention as a prominent “green” synthesis method for nanomaterials, due to its distinguishing properties when compared to solid, liquid and gas phase synthesis approaches. Furthermore, the combination of nanomaterials and plasma in biomedical applications demonstrates several synergistic effects and better treatment efficiency. A schematic diagram showing the synergistic relationship among plasmas, nanomaterials and their biomedical applications is shown in Figure 1. The use of plasmas for biomedical applications has been explored in various ways in the last few decades and have shown promising effects. The uses of nanomaterials in biomedical applications are also well known. Recently, synergistic effects of nanomaterials and cold plasmas in biomedical applications have been discovered. In this article, a review on the relationship between plasma and nanomaterials is presented. A brief description of non-thermal atmospheric pressure plasmas is included in Section 2. In Section 3, the synthesis of nanomaterials using different types of plasma is summarized. Section 4 focuses on the current advances related to the synergistic effects of plasma and nanomaterials in biomedical applications.

## 2. Overview of Non-Thermal Atmospheric Pressure Plasmas and Their Characteristics

Physical plasmas are ionized gases which generally contain electrons, ions, neutrals, excited species, electric field, reactive species, UV photons, etc. They exist naturally in the universe or can be generated within the laboratory environment in the earth. Production of plasma within the laboratories can be performed through dissociation of gas molecules with electrical energy confined between two electrodes. This type of plasma can be produced at low pressure, as well as at atmospheric pressure. Plasmas at low pressures (such as inductively coupled plasmas, plasma torches) are generated inside vacuum chambers and are much suitable for the uniform treatment of objects. They are also called thermal or quasi-equilibrium low-temperature plasmas, as the temperatures of light and heavy species are almost the same. At atmospheric pressures, plasmas (such as atmospheric pressure plasma jet discharges, dielectric barrier discharges (DBD)) could be generated by ionizing a gas between two narrow electrodes at ambient environment and no expensive vacuum equipment is required. Atmospheric pressure plasmas are also called non-thermal or non-equilibrium plasmas, as the electron temperature is much higher than ions or gas species and the temperature of gas species remains close to room temperature. Both thermal and non-thermal plasmas can be used for the synthesis of nanomaterials.

### 2.1. Non-Thermal Atmospheric Pressure Plasma Sources

Non-thermal atmospheric pressure plasmas generated at atmospheric conditions can be utilized for the synthesis of nanomaterials. Several types of plasma devices, such as DBD plasma or plasma jet, can be used for combinational treatments with nanomaterials [1]. The discharge generated in the ambient environment can be used for the modification of the surface properties of the materials through electrons, ions, excited species, reactive species, UVs, etc., generated through the plasma. An overview of non-thermal atmospheric pressure plasma sources and the reactive species generated by them is presented in this section.

#### 2.1.1. Dielectric Barrier Discharge (DBD)

**Figure 2:**
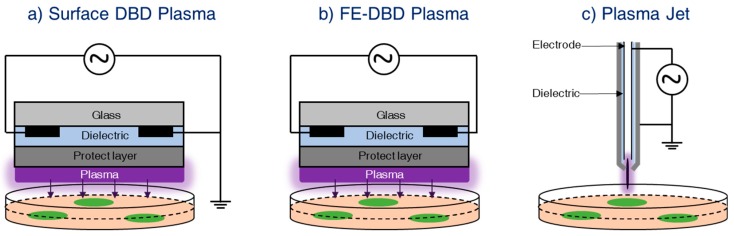
Typical structures of non-thermal DBD plasma and plasma jet devices at atmospheric pressure. (**a**) Surface DBD plasma; (**b**) FE-DBD plasma; and (**c**) plasma jet.

DBD is generated between two metal electrodes, where one electrode is connected to the high voltage power supply, and the other one is grounded. A dielectric material is placed in front of one (or both) metal electrode in order to prevent arcing. This is also known as silent or atmospheric-pressure-glow discharge [2], and was described for the first time by Ernst Werner von Siemens in 1857 [3]. Recent understandings of the physics of DBD have given rise to several improved forms of DBD-based devices. A schematic diagram of two common DBD-based devices are shown in Figure 2a,b. Both devices are constructed in a coplanar configuration, and the electrodes are fabricated above a dielectric material like glass. A protective layer made up of MgO or Al_2_O_3_ is used above the electrodes for the prevention of hydration and the promotion of long-term operation of the device. If one part of the coplanar electrode system is connected to a high-voltage power supply and the other part is grounded, it is known as surface DBD plasma (Figure 2a) [4]. Here, the material to be treated is placed below the plasma generation region. Because of the use of coplanar electrodes that are separated by a micrometer gap, the surface DBD plasma can be generated under normal atmospheric conditions (even without gas flow). On the other hand, if all the coplanar electrodes are connected to the high-voltage power supply and the target material acts as the ground electrode (with a high capacity of charge storage) such that the plasma generated in between the high voltage and the target is utilized for the modification of nanomaterial, it is known as floating electrode DBD plasma (FE-DBD) [5], as schematically shown in Figure 2b. Both types of plasma sources generate plasma over a wide area and can be used for the synthesis of nanomaterials.

#### 2.1.2. Plasma Jet

The plasma jet is an improved design of DBD. It was designed in order to extend the plasma plume at a long distance away from the discharge region, enabling the remote treatment of the sample. Extensive research works have been performed on the design of plasma jets for biomedical applications [6,7,8]. A schematic diagram of the plasma jet is as shown in Figure 2c. It consists of a high-voltage needle electrode and a ground electrode separated by a dielectric material. An inert gas flow (normally helium, argon, etc.) is maintained through the needle electrode in order to facilitate the breakdown process. Plasma is generated between the high-voltage and the ground electrodes with the help of working gases. Because of the high gas flow rate, the plasma constituents, including the short-lived reactive species, are carried towards the remote region, which is several centimeters from the discharge region. The temperature of the plasma carried to the remote region is close to room temperature and can be used for the surface modification/synthesis of nanomaterials.

### 2.2. Reactive Species Induced by Non-Thermal Atmospheric Pressure Plasmas and Their Applications

Plasma-synthesized nanomaterials can be used for various kinds of biological applications and for improving human health. The key to this application lies in the variety of reactive oxygen and nitrogen species (RONS) generated by the non-thermal atmospheric pressure plasma sources [9,10,11,12,13,14]. Plasma liquid interactions play a great role in the formation of nanomaterials as the chemistry of liquid is altered by the plasma generated RONS. The major RONS formed during plasma-liquid interactions include superoxide, hydroxyl radical, singlet oxygen, nitric oxide, ozone, hydrogen peroxide, etc. [15,16]. The densities of these species generated in plasma discharges are very high and have been tabulated in Table 1. Energetic electrons, heavy ions and UV photons present in the discharge may be responsible for enhancing the formation of these reactive species.

**Figure 3:**
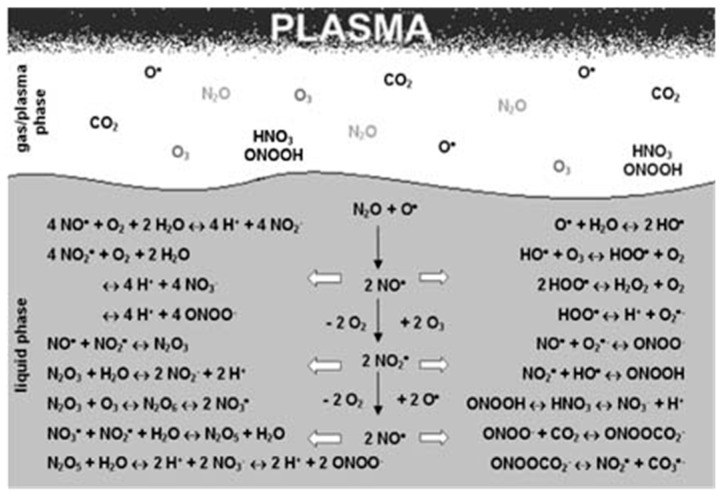
Possible reaction channels of plasma/gas–liquid interactions. With permission from Ref. [26] Copyright 2011 Wiley (Plasma Processes and Polymers 2011, 8, 904–913, DOI: 10.1002/ppap.201000099).

Few reported possible reactions for plasma–liquid interactions are given in Figure 3. There are many possibilities by which plasma-generated reactive species can have a direct or indirect effect on nanomaterial synthesis in aqueous medium. Some can have a direct effect, and some can react to form more stable long-lived reactive species, which can also have activity against biological samples. H_2_O_2_ is one of the most stable reactive species that can be generated from various other reactive species such as ·OH and superoxide [21,22,23]. Even though ·OH has a short lifetime, our group postulates that plasma-initiated UV may propagate into the aqua solution to result in the photolysis of water molecules for the production of ·OH even to or inside the cells [24]. Oehmigen et al. (2011) suggested most of the possible reactions for plasma gas/liquid interactions as shown in Figure 3. Plasma can not only be applied for surface modification, sterilization of medical equipment, antimicrobial, dermatology (including wound healing), etc. but also for cancer treatment by an endoscopic or branched organ targeting treatment technology [25].

In a recent report, Laroussi et al. demonstrated the effectiveness of a low-temperature atmospheric-pressure plasma pencil on human T-cell leukemia cells [27]. Numerous other reports have explored the efficiency of cold plasmas on various types of cancer and normal cell lines [28,29,30,31,32,33,34,35,36,37,38,39,40,41,42,43,44,45,46,47,48,49]. Various types of non-thermal plasmas offer the ability to deliver ROS directly or indirectly into living tissues, implying its feasibility as an innovative device for use in cancer therapy by endoscopic or branched organ targeting treatment technology [50,51,52]. Some types of plasma devices, such as DBD plasmas or plasma jets, can be used for the combinational treatments with nanomaterials [1]. Recently, many groups have used the plasma jet device in combination with nanomaterials for antimicrobial and anticancer activities.

**Figure 4:**
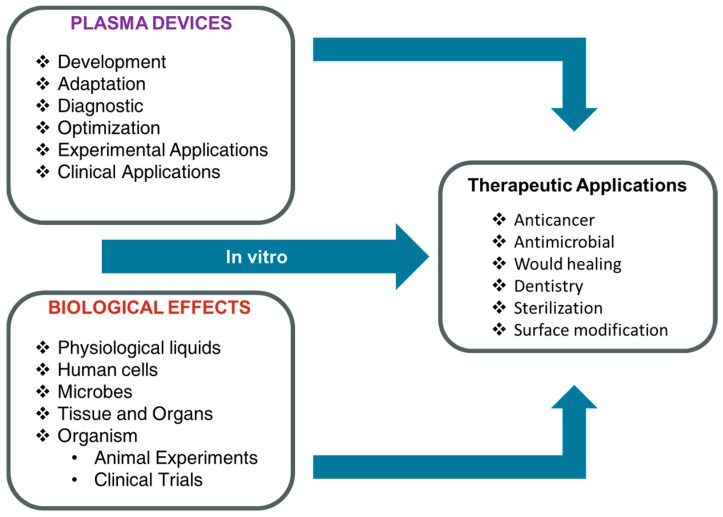
Plasma as reliable and safe therapeutic device for biomedical applications. For safe plasma clinical application, plasma devices and in vitro biological effects must be optimized.

Although, a comparatively new field in medicine, the use of non-thermal atmospheric plasmas on tumor cells has attracted a great deal of attention [53]. The outcomes of several research groups have increased the hope that non-thermal plasmas could be an interesting new therapy alternative in cancer treatment. Much emerging evidence strongly indicates that cold plasma treatment could abolish the tumor cells without damaging the normal counterparts [33,54,55]. Numerous in vitro studies support the concept of the non-thermal plasma selectivity for many types of cancer cells, including breast cancer cells [55,56], melanoma cells [42,57], skin cancer cells [58], colon carcinoma cells [58], ovarian cancer cells [59], glioblastoma cells [60,61,62], and blood cancer cells [63]. A better understanding of biological pathways or mechanisms of plasma-mediated apoptosis, including its selectivity, will help to introduce it as a novel tool for application in anticancer treatment as illustrated in Figure 4.

## 3. Application of Plasma for the Synthesis and Modification of Nanomaterials

Nanomaterials for biomedical applications have been receiving a great deal of attention due to their unique physical and chemical properties. Noble metals, transition metals and alloys, semiconductors and carbon-based nanomaterials are among the most prominent materials in biomedical applications, including cancer therapy, bioimaging, drug delivery, tissue engineering, etc. [64,65,66,67,68,69]. Nevertheless, the most common strategy for nanomaterials fabrication is chemical synthesis, which involves dangerous and toxic chemical compounds as oxidizing/reducing agents. Thus, there is a need to develop alternative “green” approaches to control the toxicity of nanomaterials.

**Figure 5:**
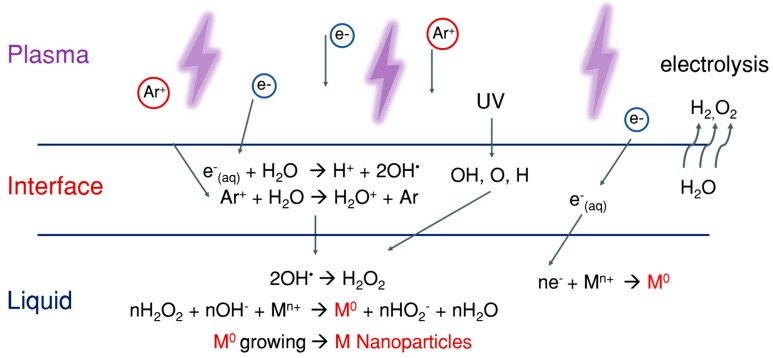
A schematic illustration of the metal ion reduction, diffusion and nucleation at the plasma-liquid interface. The reduction reactions can occur in liquid medium by either solvated plasma-induced electron or plasma induced reactive species.

Plasma technology has been widely used in the fabrication of nanomaterials [70], especially low-pressure techniques such as Plasma-Enhanced Chemical Vapor Deposition (PE-CVD) and Sputtering. In contrast with low-pressure plasma, atmospheric plasma technology also can be used for the synthesis of nanomaterials while functioning in ambient room condition. Due to the advantages of operating in air, atmospheric plasma can produce a high quantity of RONS, which can directly reduce metal ions in liquid to form metal nanoparticles (NPs), without the presence of any additional reducing agents (Figure 5). Thus, this method is also considered a “green” method for the fabrication of nanomaterials towards applications in biomedical research. Herein, we summarize recent advances on the fabrication of nanomaterials by plasma.

### 3.1. Noble Metal Nanomaterials

Noble metals like Au, Ag, Pt and Pd possess high resistance to oxidation and corrosion in moist conditions and have been used in jewelry and currency since ancient times. In contrast to the bulk materials, the noble metals in nanometer size demonstrate unique physical properties, including the surface plasmon resonance phenomenon. Surface plasmon is an evanescent electromagnetic wave at the metal surface caused by free electron oscillation under light irradiation. Due to this property, Au and Ag NPs have been utilized in a great number of applications, such as Surface-Enhanced Raman Scattering, biosensors for biological processes, nanomedicine, chemical oxidation reactions, etc. The plasma synthesis of Ag nanomaterials was first reported in 1999, through arc discharge generated over a solution of NaNO_3_ with silver electrodes; one was over the solution and another was immersed in the solution [71]. In 2005 Koo et al. reported the formation of PtNPs by exposing to H_2_/He gas plasma on the solution of H_2_PtCl_6_ [72]. Since then, materials scientists have focused on the production of NPs by using plasma over liquid system.

As pioneers in the field of plasma synthesis, Sankaran and his group attributed the Au and Ag NP formation to the electron reduction Au and Ag ions provided by plasma either in anodic dissolution or the pristine metal salts [73,74]. A simple microplasma setup was employed for the study, and microplasma was generated between the surface of a liquid solution and a stainless-steel capillary (0.7 mm distance) with the help of inert gas (helium) at a flow rate of 25 sccm. The internal diameter of the metal capillary was less than 1 mm, and it was connected to the ground through a 100 kΩ resistor. A high-voltage DC (up to 2 kV) was applied to a counter metal foil (Au or Ag) electrode immersed few millimeters away from metal capillary and a current of 0–5 mA sustained the microplasma. Under the plasma discharge, the metal dissolution occurred at the metal foils surface, followed by the formation of metal NPs in liquid phase. Instead of metal foils, aqueous solutions of HAuCl_4_ or AgNO_3_ with different concentrations (from 0.05 to 1 mM) can also be used as a precursor for the synthesis of Au and Ag NPs with the same experimental setup. Electrons were injected into the liquid solution from the plasma, thus inducing plasma–liquid electrochemistry for the synthesis of nanomaterials [75]. The electrons were injected in the solution and induced the reduction of Au salt, forming Au^0^ atoms, followed by the diffusion and aggregation to form NPs. The gas velocity of 25 sccm supposed to limit the interaction of the plasma with the surrounding air and study is focused on the influence of negatively charged species. With the above experimental setup, the microplasma processing was performed for 10 min, at which point the solution changed its color from transparent to purple, indicating the formation of Au NPs. This was verified by Transmission Electron Microscopy and a UV-visible absorption spectrum with resonance peak at 539 nm. The resonance peak wavelength and bandwidth depend on the size of the Au NPs [76]. The bandwidth also depends on the distribution of Au NPs. The size and distribution of Au NPs is usually determined by the concentration of HAuCl_4_ solution. The colloidal Au NPs produced in this way were short-lived after processing. The stability can vary between 6 h and several months, depending on several conditions, such as storage conditions, precursor concentration and the processing current, even without using any surfactants [77]. A quantitative study for Au and Ag NPs synthesis by microplasma was also reported recently [78]. The mechanism of dissociative electron attachment and Au NP formation was also discussed. At the counter electrode, due to very high applied voltage, a large number of positive ions (e.g., H^+^) are produced, causing a lower pH and increased conductivity. These positive ions can participate in the overall liquid chemistry or recombine with other species at the plasma–liquid interface. A range of species, including e^−^, OH^•^, OH^−^, O^−^, H^−^, etc., are produced at the plasma–liquid interface, and they can lead to dissociative Electron Attachment reactions (Figure 5). The most dominant reactive channel at the liquid interface is:

H_2_O + e^−^_gas_ → H^−^ + OH^•^ (1)

If the electrons are solvated in liquid, the subsequent reaction inside the solution will occur:

H_2_O + e^−^_aq_ → H^−^ + OH^•^ (2)

The subsequent cascaded chemistry could lead to the formation of hydrogen peroxide from either OH^•^ or OH^−^ [79]. This could happen as follows:

2OH^•^ → H_2_O_2_ (3)

2OH^•^ → H_2_O_2_ + 2e^−^_aq_ (4)

When the Au^3+^ ion precursor is added to the solution, the formation of Au^0^ metal particles in the solution will occur as:

[AuCl_4_]^3+^ +3e^−^_aq_ → Au^0^ + 4Cl^−^ (5)

Recently, Mariotti et al. demonstrated a continuous droplet flow plasma reactor for the synthesis of Au NPs. This method belongs to the most efficient and highest yielding of the reported methods for Au NPs synthesis [80]. Plasma interactions, ionic liquids, and molten salts are also very efficient approaches for nanomaterial synthesis. Ionic liquids possess several advantages over water solutions, such as low vapor pressure and non-volatility. Nanoparticles synthesized using plasma-ionic liquid systems do not require stabilizers due to their electrostatic stability. Synthesis of Au-Ag NPs by sputtering in plasma-ionic liquid systems has been reported previously [81,82]. The selection of ionic liquids based on low vapor pressure can facilitate highly efficient sputtering. In another reported work, evaporation of metal ions is also used for the synthesis of some NPs [83,84]. In this experiment, a metal wire was immersed in solution and acted as cathode while another inert Pt metal mesh used as anode. Evaporated cathode material moved to the solution surface and cooled down, finally being quenched in the solution, leading to formation of NPs. The amount and size of NPs depended on the applied voltage and electrolyte solution type. A bimetallic Ag/Pt can also be produced after 30 s of pulse plasma discharge between Ag cathode and Pt anode in solution of sodium dodecylsulfonate and sodium chloride. In addition to using chemical systems, physical sputtering by argon plasma was also used to produce Au/Ag and Au/Pd NPs. Nevertheless, the main drawback of ionic liquid-based systems is the relatively low solubility of metal ions compared to water-based systems. Another disadvantage is that ionic liquid decomposition by plasma can contaminate the nanomaterials. The reducing species produced by plasma moves more easily in water solution than ionic liquid. However, there are many possibilities in the field of ionic liquids development that can address all these critical issues in plasma-ionic liquid systems for the synthesis of nanomaterials.

### 3.2. Transition Metals and Alloys

The magnetic materials family is a large and compelling subject among biomedical researchers due to the attractive super paramagnetic property. This essential property offers many opportunities for biomedical applications, such as magnetic resonance imaging, drug delivery and biomedicine. Plasma-based systems are being considered as cost-efficient and high-speed production methods for metal NPs. For instance, carbon-encapsulated NPs demonstrated low toxicity on A549 cells, which is lung fibroblast. A simple pulse discharge plasma produced between the tips of two Fe rods has been used to synthesize the iron carbide NPs, encapsulated by graphite sheets [85,86]. Other materials, such as Fe_2_O_3_ [87] and carbon-encapsulated Co, Ni and Fe [88,89] NPs, have been fabricated from an aqueous solution of cationic surfactant and ethanol.

Many researchers have also studied inert material-coated iron oxide formation. Recently, silica-coated iron oxide was formed by using non-transferred arc plasma at atmospheric pressure, with a low degree of agglomeration [90]. In this study, there are 2 types of reactors—the hot wall reactor and the cold wall reactor—that are used to cause variations in the temperature gradient during the experimental period. In conclusion, silica-coated iron oxide NPs can be prepared by using non-transferred arc plasma at atmospheric pressure with Fe(CO)_5_ and TEOS as the precursors. The synthesized particles mainly have Fe_3_O_4_ as a core particle and are covered by an amorphous external layer with a size on the order of 100 nm for cold wall reactors and below 20 nm in the case of hot wall reactors, with uniform size distribution.

Formation of metallic oxide by plasma–liquid interactions is also a simple process because of the presence of strong oxidizing species. An AC plasma was used to produce CuO NPs [91]. The plasma was generated between the Cu filaments, and another Cu filament was immersed in NaNO_3_ solutions. The reactive species generated by plasma–liquid interaction reduced Cu ions to form CuO NPs. ZnO NPs can also be produced by plasma, which was generated between Zn wire and Pt wire mesh immersed in aqueous solution of K_2_CO_3_. The median electrical power input led to the formation of flower-like ZnO NPs.

Plasma–liquid interaction can also be used for production of alloy NPs [92,93]. Alloying of nanomaterials is one of the strategies for stabilizing NPs. FePt NPs were synthesized by using the displacement reaction. Argon plasma was used on FeCl_2_ solution to produce Fe NPs, and then PtCl_2_ was added to the molten solution, in which the Fe NPs were dissolved and produced. Pure FePt NPs were obtained by optimizing the ratio of FeCl_2_ to PtCl_2_ and plasma discharge doses. The synthesis of CoPt_3_, CoPt, SmCo NPs can also be carried out using the same method. A recent work by the Mariotti group also proved that Co_3_O_4_ NPs can also be obtained by microplasma discharged in absolute ethanol [94].

### 3.3. Non-Metal Nanomaterials

Silicon-based NPs have been used in biomedical applications because of their high specific surface areas and great chemical and mechanical stability. Great efforts have been made to synthesize this family of materials using plasma technology. In 2005, Sankaran et al. reported a continuous-flow atmospheric-pressure microdischarge reactor for photoluminescent silicon NPs [95]. Silicon NPs with a size of a few nanometers were successfully fabricated, and they exhibited photoluminescence at 420 nm with an impressive quantum yield of 30%. The particles also remained stable under ambient conditions for several months. Surface engineering of silicon nanocrystals (Si-NCs) with a special focus on photovoltaic applications was also studied by Mariotti et al. using microplasma processing [96]. Si-NCs with a diameter below 5 nm exhibited quantum confinement properties and could be applied in third-generation photovoltaic solar cells with high efficiency, low cost and limited environmental footprint. It is believed that plasma-induced liquid chemistry offers the opportunity for surface engineering and industrial scaling. Surface engineering of silicon nanocrystals was performed in ethanol solution. Nickel was used as a capillary, diamond carbon rod acted as a counter electrode, and argon gas was employed for plasma generation. After 20 min of microplasma processing, the photoluminescence properties were observed to have improved. Also, microplasma processing preserved the photoluminescence properties of Si-NCs for several days. These results have not been achieved with standard electrochemistry. A detailed study of the cause of the photoluminescence properties was performed by Fourier transform infrared analysis. This revealed that Si-NCs under microplasma processing undergo surface modification, whereby Si-H bonds and other surface terminations are removed and mostly replaced with Si-O-R terminations, promoting surface reconstruction at the outer layer. Also, the efficiency of the microplasma-processed Si-NCs device increased over the full spectral range. Recently, researchers have also made efforts to synthesize Ge and Si quantum dots by using a plasma-ionic liquid system [97].

Carbon-related nanomaterials such as multiwall carbon nanotubes, carbon nanohorns, carbon NPs, etc., are prominent advanced materials due to their exceptional physical properties, such as high conductivity, strength, stiffness, and toughness. Recently, many researchers have used plasma discharge to form carbon-related nanomaterials in liquids. Nanodiamond formation by using microplasma operated in a gaseous phase of Ar/H2/ethanol has been reported [98]. Nowadays, carbon nanotubes have been intensely and extensively researched, because they have very unique and attractive physical and electrical properties, including high electrical conductivity, extraordinary mechanical strength, and thermal and chemical stability. Therefore, it is a promising material for versatile applications in the near future. In particular, it can be applied for optical and biomedical devices, as well as various sensors. In a recent work, vertically aligned multi-walled carbon nanotubes were prepared with a diameter of less than 80 nm and uniform thickness of 16 μm, by employing microwave plasma torch with a high-speed process of only 60–120 s [99]. Carbon-encapsulated magnetic materials were also obtained by plasma as described in the previous section [88].

Table 2 summarizes current advances of several nanomaterials synthesized by plasma technologies. In the future, the main focus of plasma-induced synthesis of nanomaterials should be on the improvement of several essential factors, such as controlled synthesis, oxidation prevention, impurity and industrial scale production. More detailed studies need to implement for synthesis and diagnostic for application of nanomaterials in biomedicine and other applications.

## 4. Plasma and Nanomaterial Combination Treatment on Cells and Microbes

Non-thermal plasma can directly or indirectly kill cancer cells. Use of conjugated or un-conjugated NPs can increase the specificity and efficiency of treatment. There are many reports in which researchers use antibody-conjugated NPs to enhance cell death and reduce the cell viability of cancer cells. In future, this combination technology could become a feasible therapeutic technology against various kinds of cancers or tumors.

In cancer treatment, it is very important to induce cell death specifically in cancer tissue or mass. However, plasma cannot efficiently distinguish between normal and cancer tissue. Recently, researchers used Au NPs (which are harmless to the human body at low doses) conjugated with antibody (which targets overexpressed proteins on or in cancer cells). Choi et al. used Au NPs conjugated with antibody against epidermal growth receptor (EGFR) [45]. EGFR protein is overexposed in many types of cancer cells, more specifically in oral cancer cells. They used low doses of air plasma (9.2 J/cm^2^ for 30 s) with anti-EGFR antibody-conjugated Au NPs to selectively kill oral cancer cells. Morphology was changed from a spindle shape to a round shape, cell shrinkage and membrane rupture were observed in oral cancer cells treated with plasma plus conjugated Au NPs. However, the whole mechanism for this combination treatment is still not known, and has to be elucidated in the future.

The research group of Prof Michael Keider and Jonathan H. Sherman demonstrated the synergistic effect of Au NPs and cold plasma on glioblastoma (brain cancer) cancer cells [100]. They revealed that the synergistic effect between Au NPs and atmospheric pressure cold plasma is dependent on the concentration of Au NPs. Au NPs significantly increase cell death by 30% in glioblastoma cell line with the same plasma dosage. They concluded that intracellular reactive oxygen species accumulation results in the oxidative stress, which further changes the intracellular pathways, and also increases damage to protein, DNA and lipid molecules. These studies showed that synergy has great potential to improve cancer therapy and reduce the harm to normal cells.

Researchers also targeted NEU (human epidermal growth factor receptor 2) protein, which is frequently overexpressed on the cell membrane of skin cancer (melanoma) cells. They used anti-NEU antibody-conjugated Au NPs in this recent study. These nanoparticles were selectively uptaken by skin cancer cells rather than normal keratinocyte. Both cancers, as well as normal cells, were treated by non-thermal atmospheric pressure plasma after the addition of conjugated Au NPs. This combination treatment selectively enhanced the death rate of skin cancer cells significantly rather than normal keratinocyte cells. This selective skin cancer cell death is may be due to selective destruction of NEU protein and a downstream effect of NEU [101].

Recently, non-thermal atmospheric pressure plasma with Au NPs was also used for killing microbes. Non-thermal plasma efficiently kills oral bacteria on agar plates; however, it has less effect on the tooth surface. Therefore, researchers used 30-nm Au NPs to enhance the killing effect of non-thermal on oral bacteria (*Streptococcus mutans*) on the tooth. Non-thermal plasma treatment alone decreased viability by 5 log of *S. mutans* in vitro; however, plasma treatment of bacteria on tooth surface exhibited only a 3-log reduction in viability. When they added Au NPs to the bacteria, plasma treatment also showed a 5-log reduction in the viability of bacteria, while Au NPs alone did not show any antibacterial effect. The morphological examination by transmission electron microscopy showed that plasma treatment only perforated the bacterial cell walls; however, combination treatment with plasma and Au NPs caused significant cell damage, causing loss of intracellular components from many bacterial cells [102].

Our group has also reported the synergistic effect of PEG-coated Au NPs (PEG-Au NPs) and non-thermal plasma on epithelial-mesenchymal transition (EMT) and the maintenance of cancer stem cells (CSC) on solid cancer cells. The results showed that co-treatment with PEG-GNP and non-thermal plasma inhibited growth in cancer cells by altering the PI3K/AKT signaling axis. This non-thermal plasma and PEG-Au NP co-treatment reversed EMT in tumor cells by altering signaling proteins, resulting in the upregulation of epithelial markers such as E-cadherin and down-regulation of N-Cadherin, Slug and Zeb-1. It was also shown that this co-treatment also inhibited tumor growth by decreasing mesenchymal markers in tumor xenograft mice models. This kind of combination treatment also inhibited sphere formation and the self-renewal capacity of glioma-like stem cells [103,104].

In another recent report, the synergistic cytotoxicity of Au NPs and non-thermal plasma showed enhanced endocytosis and trafficking to the lysosomal compartment as well as temporarily increased membrane permeability. This report contributes knowledge to the mechanism of combination effects of non-thermal plasma and NPs and indicates a technology for possible drug delivery systems. It is demonstrated that the rates of Au NPs uptake and total amount accumulated in solid cancer cells are significantly enhanced after exposure to 75 kV non-thermal plasma generated by DBD. Chemical effects induced by direct and indirect exposure to non-thermal plasma appear the dominant mediator of enhanced uptake [105]. They also showed that Au NPs and non-thermal co-treatment caused many divots across the glioma cell membrane, making it more porous. In contrast, there was no significant effect or NP uptake in astrocyte (E6/E7) cells, and there was no change in the cell membrane morphology. These studies prove that plasma-based nano-drug delivery technology is safe and effective against solid tumors [106].

To maximize the preferential killing of melanoma cells non-thermal plasma is used with Au NPs tagged with antibodies targeting phosphorylated FAK (p-FAK). Combined treatment also showed the minimum effect against HaCaT keratinocyte cells. After co-treatment on melanoma cells, signaling molecules such as FAK, p-paxillin, and NEU were reduced with treatment. Therefore, it is suggested that these kinds of co-treatment strategies are effective and selective against melanomas [107]. Recently core-shell NPs were synthesized via co-axial electrospraying. Biocompatible poly (lactic-*co*-glycolic acid) was used as the polymer shell to encapsulate anti-cancer treatment. In vitro studies demonstrated the synergistic effect of these core-shell NPs and non-thermal plasma for inhibition of breast cancer cell growth when compared to each treatment alone. Non-thermal plasma induced down-regulation of metastasis-related gene expression (VEGF, MTDH, MMP9, and MMP2) and facilitated drug-loaded nanoparticle uptake, which could be an important breakthrough minimizing drug resistance, a major problem in cancer chemotherapy [108].

Iron NPs 50 nm in size were also used for combination treatment with non-thermal plasma against human breast adenocarcinoma cells. Findings showed that the combination of plasma and iron NPs inhibited the viability of cancer cells significantly. Molecular analysis demonstrated that the combination treatment induced shifting the *BAX/BCL-2* ratio in favor of apoptosis [109]. Table 3 summarizes recent updates on plasma and nanomaterials combination for cancer treatment.

In addition, cold plasmas are widely used as treatment tools for biomaterial surfaces, such as polymers, metals, alloys, ceramics, and composites [112]. The plasma treatment is capable to activate or functionalize the material surfaces and also produces several exclusive properties compared to other chemical and physical methods. Due to the low penetration capability, plasma can only be able to modify very few thin layers of the surface with a thickness of a hundred nanometers. Thus, the surface properties can be altered, in order to enhance the biocompatibility and bio-functionalization, which are vital parameters towards practical applications of synthetic biomaterials. In general, plasma-modified biomaterials possess great potential for the applications in dentistry, tissue engineering or drug delivery. For instance, metal NPs with antimicrobial or bactericide properties can be incorporated or coated on the surfaces of implants by plasma modification techniques. Plasma treatment also can change the surface roughness and increase the hydrophilicity of biodegradable polymers, thus improve the cell affinity and cell adhesion of these materials. Moreover, plasma surface modification can promote the attachment, loading, and release of drug molecules in porous biomaterials.

Recently, Rosales et al. have reported the effect of plasma surface modification on antibacterial properties of Cu NPs [113]. The surface of Cu NPs was functionalized with polyacrylic acid, polyacrylonitrile, or polymethyl methacrylate by utilizing the radiofrequency plasma reactor to enhance the biocompatibility. During the plasma polymerization, the simultaneous removal of CuO upper layers on the surface of Cu NPs was observed. The polymer layers also acted as protecting layers, preventing Cu NPs being oxidized. The coating polymer films on Cu NPs were thin enough for the copper ions to easily diffuse through the coating layer and subsequently interact with bacteria. The antibacterial properties of the Cu NPs were not significantly affected by the plasma modification. They demonstrated outstanding antibacterial property against Gram-positive bacteria (*P. aeruginosa*). The plasma polymerization-coated Cu NPs also possessed significantly lower toxicity to normal human cells as compared to pristine copper NPs.

Titanium biomaterials are frequently used in medical and dental sciences. Titanium dental implants have promising potentials as the replacements of missing teeth. However, there are chances of failure in dental implants after surgeries in extraction sockets. Atmospheric pressure plasma treatment can be used to soft tissue around titanium dental implant abutments surface without causing topological changes. The topology and chemistry of titanium implants are important factors for osseointegration. A preliminary research work in this field studied the cellular attachment and differentiation of titanium nanotubes treated by air or nitrogen non-thermal atmospheric pressure plasma jets [114]. After plasma treatment the ostogenic properties were improved without any change in topographical morphology. In addition, plasma treatment of nanotubes further increased hydrophilicity, as well as modifying the surfaces. It was demonstrated that cellular activity and in vitro osseointegration were significantly enhanced on plasma-treated titanium nanotube.

Another example is Poly(lactide-*co*-glycolide) or PLGA NPs, which can be applied for sustained delivery of proteins for nervous tissue repair. However, sterilization of protein-loaded PLGA is a challenging task, due to the possibility of reducing the activity of proteins. Recently, researchers from Drexel University reported sterilization of protein-loaded PLGA NPs by using indirect plasma treatment [115]. They used plasma-treated water and PBS for sterilization of NPs and proteins. Plasma-treated water showed no morphological changes to the NPs. They concluded that treatment of particles in plasma-treated PBS is effective for decontaminating particles, as well as in maintaining protein activity; thus, is possible to apply it for nervous tissue repair therapy.

## 5. Summary and Future Prospectives

Great efforts have been carried out over the last few decades focusing on the synergistic effects of non-thermal plasma and nanomaterials towards applications in biomedical science. The combination of these two emerging research fields typically refers to two different strategies. The first is the synthesis of nanomaterials using non-thermal plasma, which has been proved as a simple, effective, low-cost and, furthermore, clean method. It can eliminate the consumption of toxic chemical components during the fabrication of nanomaterials, which are typically used in other conventional methods, such as chemical and physical synthesis technologies. Thus, the toxicity of plasma-synthesized nanomaterials is relatively lower, making them become more appropriate for biomedical practical applications. Depending on the structures of the nanomaterials, the RONS induced by plasma can be encapsulated inside the materials; hence, their live-time and travel distance will increase. These two parameters are vital problems that non-thermal plasma technology has been dealing with, in order to enhance effectiveness. The second is the combination of plasma and post-synthesized nanomaterials. Under non-thermal plasma treatment, the cell membranes can be open or enlarged, therefore increasing the cell permeation. Consequently, nanomaterials can easily penetrate through cell membranes. In addition, the low selectivity property of non-thermal plasma can be compensated by nanomaterials, which are generally functionalized for specific targets.

This review briefly summarizes recent progress in the combination of plasma and nanomaterials. Important challenges remain for the utilization of this interdisciplinary field in the future. The understanding of plasma and nanomaterials interactions, in both synthesis and post-synthesis are still inadequate. Due to the presence of a variety of species, electric field and radiation, non-equilibrium chemistry processes between plasma and nanomaterials are exceptionally complex and difficult to control. The homogeneity of the desired nanostructures by plasma synthesis is limited, compared to other methods. Thus, further studies are required to understand the natures and extending the use of plasma and nanomaterials combination, not only limited to biomedical, but also many other biological applications. In addition, plasma-assisted nano-drug delivery systems are potential subjects for future biomedical researches. This advanced technology can be utilized in clinical and aesthetic purposes.

## Figures and Tables

**Table 1:** Typical relative concentrations of various charged and neutral species generated by non-thermal DBD plasma in gas phase [17,18,19,20].

Plasma Generated Species	Density (cm^−3^)
Superoxide (O_2_^−^)	10^10^–10^12^
Hydroxyl (OH^•^)	10^15^–10^17^
Hydrogen Peroxide (H_2_O_2_)	10^14^–10^16^
Singlet Oxygen (^1^O_2_)	10^14^–10^16^
Ozone (O_3_)	10^15^–10^17^
Nitric Oxide (NO)	10^13^–10^14^
Electrons (e^−^)	10^9^–10^11^
Positive ions (M^+^)	10^10^–10^12^

**Table 2:** Summary of nanomaterials synthesized by plasma technologies.

Materials	Methods	Average Size	References
Ag Nanowire	Arc Plasma	5–15 nm (diameter) <100 nm length	[71]
Pt NPs	RF Plasma	2 nm	[72]
Au NPs, Ag NPs	Microplasma	8 nm–10 nm	[73,74,77,78]
Au NPs	Microplasma	4.4 nm	[80]
Au NPs	Sputter	5.5 nm	[81]
Au-Ag Alloy	Sputter	2.6–6.0 nm	[82]
Ag Nanopowder	Wire explosion	20-200 nm	[83]
Au, Ag, Ti, Ni Nanoball	Plasma electrolysis	10 nm	[84]
FeC NPs	Plasma in liquid ethanol	5–600 nm	[85]
FeC Nanocapsule	Plasma in liquid ethanol	10–20 nm	[86]
Fe_3_O_4_	Pulsed Plasma in liquid	19 nm	[87]
Fe NPs	Pulsed Plasma in liquid	35 nm	[88]
Ni NPs	Pulsed Plasma in liquid	26 nm	[88]
Co NPs	Pulsed Plasma in liquid	20 nm	[88]
Fe@C NPs	Pulsed Plasma in liquid	32 nm	[89]
Ni@C NPs	Pulsed Plasma in liquid	40 nm	[89]
Fe_3_O_4_@Si	Arc Plasma	20 nm	[90]
CuO nanorods	Arc Plasma	14–16 nm	[91]
Cu NPs	Arc Plasma	30–50 nm	[91]
Cu_2_O NPs	Arc Plasma	4–10 nm	[91]
FePt NPs	Microplasma	Less the 100 nm	[92]
Co_3_O_4_ NPs	Microplasma	2–5 nm	[94]
Si NPs	Microplasma	1–3 nm	[95]
Nanodiamond	Microplasma	3 nm	[98]
Multiwalled-Carbon Nanotubes	Microwave Plasma	80 nm (diameter)	[99]

**Table 3:** Recent updates on plasma and nanomaterial combination treatment against cancers.

Published Year	Cancer Type	Plasma Device	Nanomaterial	Reference
2014	Glioblastoma	Plasma jet	Au NPs	[100]
2015	Melanoma	Surface type air plasma	Anti-NEU-Au NPs	[101]
2017, 2016	Glioblastoma	Surface DBD air plasma	PEG-Au NPs	[103,104]
2018	Glioblastoma	DBD plasma	Au NPs	[105]
2015	Glioblastoma	Plasma jet	Au NPs	[106]
2017, 2009	Melanoma	DBD Plasma	Anti-FAK-Au NPs	[107,35]
2016	Breast Cancer	Cold atmospheric plasma	Fluorouracil loaded core-shell NPs	[108]
2016	Breast Cancer	Plasma jet	Iron NPs	[109]
2015	Colorectal Cancer	Plasma jet	Au NPs	[110]
2017	Lung Cancer	DBD plasma	Epidermal growth factor conjugated Au NPs	[111]

## Abbreviations

List of repeated acronyms:

UV	ultra-violet
DBD	dielectric barrier discharge
FE-DBD	floating electrode dielectric barrier discharge
RONS	reactive oxygen and nitrogen species
ROS	reactive oxygen species
OH	hydroxyl radical
O_2_^−^	super oxide
H_2_O_2_	hydrogen peroxide
^1^O_2_	singlet oxygen
O_3_	ozone
E^−^	electron
M^+^	positive ion
NPs (Au NPs, Cu NPs, Fe NPs, etc.)	Nanoparticles (Gold nanoparticles, Copper nanoparticles, Iron nanoparticles, etc.)
NCs	nanocrystals
Ar	argon
EMT	epithelial-mesenchymal transition
PEG-GNP	Polyethyleneglycol coated gold Nanoparticles
